# Supplementary material for: Modeling glioblastoma heterogeneity as a dynamic network of cell states
Source: Mol Syst Biol. 2021 Sep 16;17(9):e10105. doi: 10.15252/msb.202010105 (PMC8444284; doi:10.15252/msb.202010105)
Supplement: Supplementary file 6 — Source Data for Figure 5 [file MSB-17-e10105-s004.zip › Figure5A_sourcedata/GSEA_3071/nosigdb_stateV.GseaPreranked.1622795159954/OLSEN_MESENCHYMAL.html]

Details for gene set OLSEN\_MESENCHYMAL[GSEA]

|  || Dataset | state43071 |
| Phenotype | NoPhenotypeAvailable |
| Upregulated in class | na\_neg |
| GeneSet | OLSEN\_MESENCHYMAL |
| Enrichment Score (ES) | -0.75231755 |
| Normalized Enrichment Score (NES) | -3.8635654 |
| Nominal p-value | 0.0 |
| FDR q-value | 0.0 |
| FWER p-Value | 0.0 |
Table: GSEA Results Summary

  

Fig 1: Enrichment plot: OLSEN\_MESENCHYMAL      
 Profile of the Running ES Score & Positions of GeneSet Members on the Rank Ordered List

  

| PROBE | GENE SYMBOL | GENE\_TITLE | RANK IN GENE LIST | RANK METRIC SCORE | RUNNING ES | CORE ENRICHMENT || 1 | LHFPL6 |  |  | 118 | 1.331 | -0.0074 | No |
| 2 | LIMA1 |  |  | 264 | 1.098 | -0.0227 | No |
| 3 | COL1A2 |  |  | 1028 | 0.614 | -0.1617 | No |
| 4 | ITM2B |  |  | 1244 | 0.547 | -0.1967 | No |
| 5 | EGR1 |  |  | 1691 | 0.438 | -0.2770 | No |
| 6 | C1R |  |  | 1696 | 0.437 | -0.2728 | No |
| 7 | ANXA1 |  |  | 2165 | 0.366 | -0.3582 | No |
| 8 | EEF1D |  |  | 2307 | 0.349 | -0.3813 | No |
| 9 | FOS |  |  | 2529 | 0.319 | -0.4199 | No |
| 10 | PRRX1 |  |  | 2674 | 0.302 | -0.4441 | No |
| 11 | TIMP3 |  |  | 2826 | 0.286 | -0.4697 | No |
| 12 | IFITM2 |  |  | 3585 | -0.312 | -0.6112 | No |
| 13 | GSN |  |  | 3695 | -0.331 | -0.6283 | No |
| 14 | VIM |  |  | 3830 | -0.354 | -0.6500 | No |
| 15 | JUNB |  |  | 4027 | -0.394 | -0.6830 | No |
| 16 | PLAC9 |  |  | 4197 | -0.437 | -0.7104 | No |
| 17 | FSTL1 |  |  | 4262 | -0.460 | -0.7174 | No |
| 18 | CD99 |  |  | 4339 | -0.484 | -0.7264 | No |
| 19 | B2M |  |  | 4341 | -0.485 | -0.7211 | No |
| 20 | IFITM3 |  |  | 4424 | -0.509 | -0.7310 | No |
| 21 | VCAN |  |  | 4491 | -0.537 | -0.7375 | No |
| 22 | CCDC80 |  |  | 4510 | -0.546 | -0.7347 | No |
| 23 | COL14A1 |  |  | 4517 | -0.548 | -0.7296 | No |
| 24 | TPT1 |  |  | 4579 | -0.576 | -0.7347 | No |
| 25 | UACA |  |  | 4621 | -0.604 | -0.7357 | No |
| 26 | IGFBP7 |  |  | 4622 | -0.604 | -0.7288 | No |
| 27 | RPS25 |  |  | 4663 | -0.625 | -0.7293 | No |
| 28 | LGALS1 |  |  | 4784 | -0.699 | -0.7443 | Yes |
| 29 | S100A13 |  |  | 4791 | -0.705 | -0.7375 | Yes |
| 30 | MT2A |  |  | 4799 | -0.714 | -0.7307 | Yes |
| 31 | IGFBP6 |  |  | 4800 | -0.715 | -0.7225 | Yes |
| 32 | PPIC |  |  | 4802 | -0.716 | -0.7146 | Yes |
| 33 | NPC2 |  |  | 4841 | -0.750 | -0.7133 | Yes |
| 34 | THY1 |  |  | 4874 | -0.781 | -0.7105 | Yes |
| 35 | ZFP36L1 |  |  | 4929 | -0.843 | -0.7112 | Yes |
| 36 | CST3 |  |  | 4951 | -0.868 | -0.7054 | Yes |
| 37 | CALD1 |  |  | 4960 | -0.877 | -0.6969 | Yes |
| 38 | IGFBP5 |  |  | 4978 | -0.897 | -0.6900 | Yes |
| 39 | RRBP1 |  |  | 4981 | -0.898 | -0.6801 | Yes |
| 40 | NNMT |  |  | 5004 | -0.942 | -0.6736 | Yes |
| 41 | EMP1 |  |  | 5023 | -0.979 | -0.6659 | Yes |
| 42 | SERPINH1 |  |  | 5059 | -1.050 | -0.6606 | Yes |
| 43 | FTL |  |  | 5072 | -1.077 | -0.6507 | Yes |
| 44 | COL5A2 |  |  | 5112 | -1.189 | -0.6446 | Yes |
| 45 | CAVIN1 |  |  | 5116 | -1.195 | -0.6315 | Yes |
| 46 | NUPR1 |  |  | 5123 | -1.217 | -0.6188 | Yes |
| 47 | TIMP1 |  |  | 5134 | -1.251 | -0.6065 | Yes |
| 48 | LMNA |  |  | 5141 | -1.277 | -0.5931 | Yes |
| 49 | GADD45B |  |  | 5155 | -1.335 | -0.5803 | Yes |
| 50 | SERPINF1 |  |  | 5158 | -1.351 | -0.5653 | Yes |
| 51 | S100A6 |  |  | 5183 | -1.473 | -0.5531 | Yes |
| 52 | PRSS23 |  |  | 5189 | -1.489 | -0.5371 | Yes |
| 53 | S100A11 |  |  | 5196 | -1.536 | -0.5208 | Yes |
| 54 | COL4A2 |  |  | 5198 | -1.545 | -0.5034 | Yes |
| 55 | BGN |  |  | 5216 | -1.654 | -0.4878 | Yes |
| 56 | IGFBP4 |  |  | 5226 | -1.725 | -0.4699 | Yes |
| 57 | DCN |  |  | 5233 | -1.798 | -0.4505 | Yes |
| 58 | TPM2 |  |  | 5247 | -1.967 | -0.4306 | Yes |
| 59 | PCOLCE |  |  | 5248 | -1.999 | -0.4078 | Yes |
| 60 | MGP |  |  | 5252 | -2.037 | -0.3852 | Yes |
| 61 | COL6A2 |  |  | 5265 | -2.276 | -0.3616 | Yes |
| 62 | COL4A1 |  |  | 5266 | -2.279 | -0.3356 | Yes |
| 63 | CTHRC1 |  |  | 5270 | -2.394 | -0.3089 | Yes |
| 64 | COL5A1 |  |  | 5274 | -2.455 | -0.2815 | Yes |
| 65 | LUM |  |  | 5277 | -2.507 | -0.2533 | Yes |
| 66 | MYL9 |  |  | 5280 | -2.643 | -0.2236 | Yes |
| 67 | TAGLN |  |  | 5281 | -2.669 | -0.1932 | Yes |
| 68 | COL6A1 |  |  | 5283 | -2.747 | -0.1621 | Yes |
| 69 | FN1 |  |  | 5287 | -2.866 | -0.1300 | Yes |
| 70 | COL3A1 |  |  | 5292 | -3.630 | -0.0894 | Yes |
| 71 | COL6A3 |  |  | 5293 | -3.722 | -0.0470 | Yes |
| 72 | COL1A1 |  |  | 5294 | -4.144 | 0.0002 | Yes |
Table: GSEA details [plain text format]

  

Fig 2: OLSEN\_MESENCHYMAL: Random ES distribution      
 Gene set null distribution of ES for **OLSEN\_MESENCHYMAL**
